# Real-Time Rotation Estimation Using Histograms of Oriented Gradients

**DOI:** 10.1371/journal.pone.0092137

**Published:** 2014-03-24

**Authors:** Blaž Bratanič, Franjo Pernuš, Boštjan Likar, Dejan Tomaževič

**Affiliations:** 1 Sensum, Computer Vision Systems, Ljubljana, Slovenia; 2 Laboratory of Imaging Technologies, Faculty of Electrical Engineering, University of Ljubljana, Ljubljana, Slovenia; College of Mechatronics and Automation, National University of Defense Technology, China

## Abstract

This paper focuses on real-time rotation estimation for model-based automated visual inspection. In the case of model-based inspection, spatial alignment is essential to distinguish visual defects from normal appearance variations. Defects are detected by comparing the inspected object with its spatially aligned ideal reference model. Rotation estimation is crucial for the inspection of rotationally symmetric objects where mechanical manipulation is unable to ensure the correct object rotation. We propose a novel method for in-plane rotation estimation. Rotation is estimated with an ensemble of nearest-neighbor estimators. Each estimator contains a spatially local representation of an object in a feature space for all rotation angles and is constructed with a semi-supervised self-training approach from a set of unlabeled training images. An individual representation in a feature space is obtained by calculating the Histograms of Oriented Gradients (HOG) over a spatially local region. Each estimator votes separately for the estimated angle; all votes are weighted and accumulated. The final estimation is the angle with the most votes. The method was evaluated on several datasets of pharmaceutical tablets varying in size, shape, and color. The results show that the proposed method is superior in robustness with comparable speed and accuracy to previously proposed methods for rotation estimation of pharmaceutical tablets. Furthermore, all evaluations were performed with the same set of parameters, which implies that the method requires minimal human intervention. Despite the evaluation focused on pharmaceutical tablets, we consider the method useful for any application that requires robust real-time in-plane rotation estimation.

## Introduction

Quality control of an industrial process covers all aspects that influence the quality of a product [1]. It is based on measuring and assessing process parameters such as temperature and pressure as well as various product characteristics such as shape, hardness, composition, and visual appearance. Visual appearance can be important both for functional and aesthetic reasons and for compliance with statutory and regulatory requirements. Due to imperfect production processes, a compliant visual appearance is usually assured by a subsequent visual quality inspection [2]–[4].

Visual inspection is performed either manually by trained personnel or automatically by inspection machines. Manual inspection is slow, tedious, and subjective. It was reported [4] that human inspectors are prone to classify inspected objects as defective only to satisfy a rejection quota. Nevertheless, the high flexibility of a manual inspection still makes it a viable option for small production batches. Automatic inspection, by contrast, produces fast, objective, and reproducible results but requires a sophisticated system consisting of mechanical manipulation, acquisition, registration, and analysis. Registration and analysis are difficult, because products can vary in size, shape, and surface complexity. Moreover, due to surface defects being visible only under directed illumination, registration and analysis must cope with rotation dependent object surface appearance.

Analysis only becomes tractable by the integration of *a priori* knowledge of the object, i.e., a reference model [3], [5]. Analysis is then performed by comparing the inspected object with its spatially aligned, ideal reference model. Rotation estimation is crucial for the inspection of rotationally symmetric objects where mechanical manipulation is unable to ensure correct object rotation.

**Figure 1:**
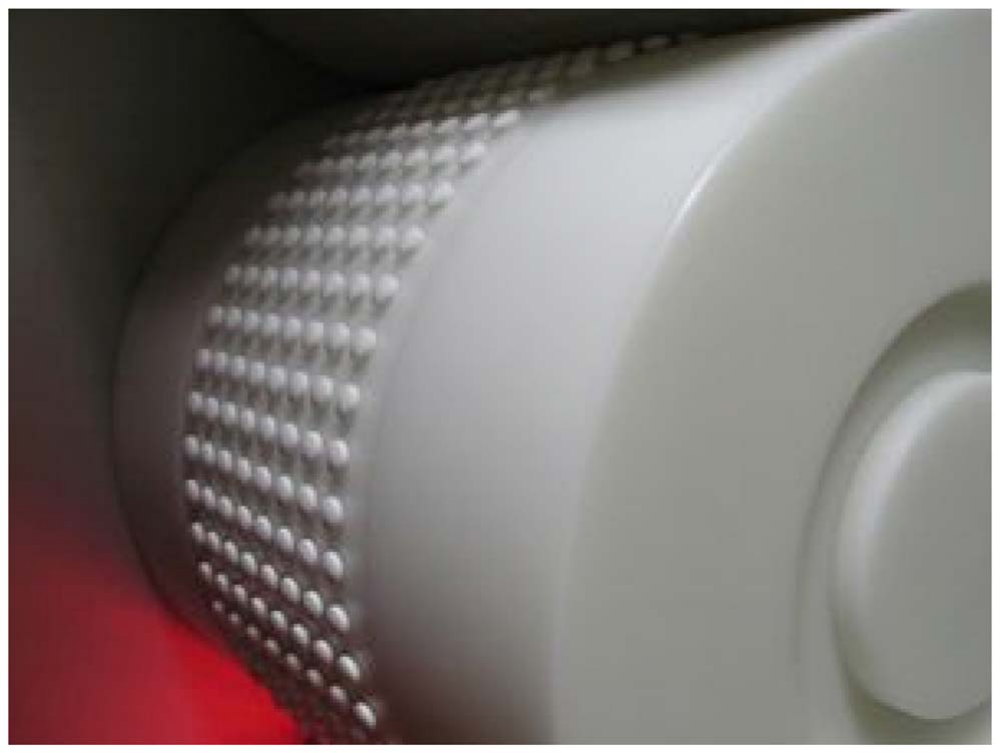
Mechanically constrained inspected products on an automated inspection machine.

We propose a method for model-based real-time rotation estimation. Rotation estimation uses an ensemble of nearest-neighbor estimators, where each estimator corresponds to a stationary spatially local region at various object rotations. Estimations from all estimators are weighted, based on the angle for which the estimator votes. Weighted estimations are then accumulated in a vote accumulator. The estimated angle is the angle with the maximum number of votes. Further, we describe the estimator's weight estimation and propose a method to improve estimators from unlabeled training samples. The method was evaluated on several datasets of pharmaceutical tablets. At inspection, the tablets are mechanically constrained on a rotating drum, as shown in Figure 1. Rotation estimation of pharmaceutical tablets is a challenging task because of normal intra- and inter-tablet variability, which occurs due to imperfect production processes, the required generality of the method – it should work for a variety of shapes, imprints, and surface appearances – and speed requirements. A typical inspection machine inspects 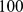 tablets per second.

Although the evaluation is focused on pharmaceutical tablets, we consider the method useful for any application that requires robust real-time in-plane rotation estimation.

## Related Work

Špiclin et al. [6] proposed three registration methods for in-plane rotation estimation: Direct Pixel Matching (DPM), Principal Axes Matching (PAM), and Circular Profile Matching (CPM). Direct Pixel Matching evaluates a similarity measure (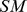) between the reference and a sample image. 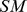 is a normalized cross-correlation of the rotating reference image and a sample image. The rotation angle 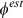 is estimated by maximizing the 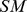 between the two: 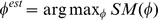. The method directly matches the pixels in 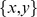 space; thus the SM must be evaluated for each rotation angle individually. It therefore requires a rotation of the reference image. Each rotation includes an interpolation of the whole image, which induces a large performance penalty.

Circular Profile Matching, on the other hand, is based on the extraction and alignment of circular profiles. A circular profile is a 1-D function 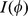 obtained by integration of intensity values of a 2D image 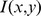 within a ring centered at the tablet center:
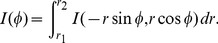
(1)


The rotation angle is estimated by a 1-D cross correlation of the reference's and the sample's circular profiles. Compared to the DPM, performance is improved by reducing the problem to one dimension.

Principal Axes Matching estimates the rotation between the reference and a sample image by matching the angles of the principal axes [7]. The principal axes are obtained on the assumption that an image 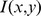 represents a density function 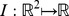. Principal axes are the eigenvectors of the covariance matrix 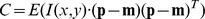, where 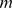 denotes the image center 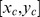 and 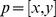. The angle between the reference and sample image is obtained directly from the angles of the principal axes between the two images. Principal axes have a residual 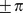 sign ambiguity. To determine the rotation with principal axes reliably and accurately, matched images must also contain a distinctive shape asymmetry, and additional validation must be performed to resolve the 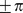 ambiguity. Špiclin et al. extensively evaluated the described methods and concluded that only the CPM method is suitable for rotation estimation of pharmaceutical tablets.

In general, prior work on rotation estimation is either sensitive to outliers due to non-robust global similarity measure [8], requires extensive parameter tuning, assumes rotationally symmetric illumination or does not run in real time.

## Method

The key idea of the proposed method is to exploit the rotation dependence of HOG features to construct a representation of an object in a feature space for all rotation angles. Features are calculated on a dense grid of spatial regions, which tile a detection window. The detection window is determined in a preprocessing step from the boundaries of the segmented area [9]. Each spatial region corresponds to a separate reference set that contains a region appearance – in HOG feature space – at all rotation angles. Angle estimation is performed with an ensemble of nearest-neighbor (in Euclidean space) estimators, where each estimator individually votes for the resulting angle.

**Figure 2:**
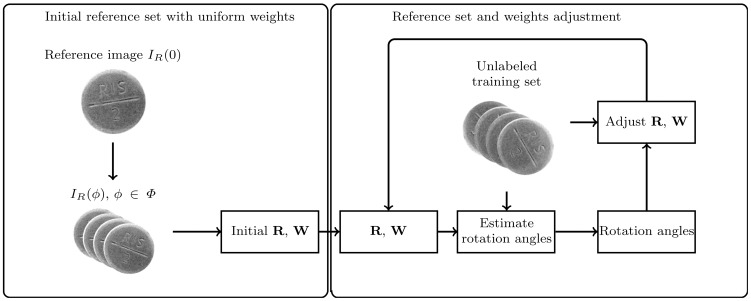
Training process. An initial weight and reference set estimations (left) are iteratively refined with a set of unlabeled training images (right). 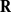 and 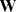 denote reference and weights set respectively.

The method is divided into two phases: the training and the process phase. In the training phase, (Figure 2) reference sets describing object appearance on a valid rotation interval are constructed. A valid rotation interval is set by the user and determines valid object rotations (usually on the interval 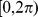). The rotation interval is discretized into a set of valid rotation angles 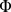 using a predefined discretization step 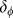. Each reference set contains feature vectors describing the corresponding spatial regions at all valid rotation angles. Reference sets are initially constructed by rotating a single reference image and are then adjusted with a set of unlabeled training images. The unlabeled training set is also used to determine weights for each spatial region.

In the process phase, the constructed reference sets and estimated weights are used for angle estimation. Estimation is performed with an ensemble of nearest-neighbor estimators where each estimator votes for the resulting angle.All votes are weighted and accumulated. The resulting angle is the angle that accumulated most votes.

We begin with a brief description of Histograms of Oriented Gradients and then describe the construction of initial reference sets, their iterative adjustment, and a weight estimation scheme. We conclude with the description of a weighted voting-based rotation estimation.

### Histograms of Oriented Gradients

**Figure 3:**
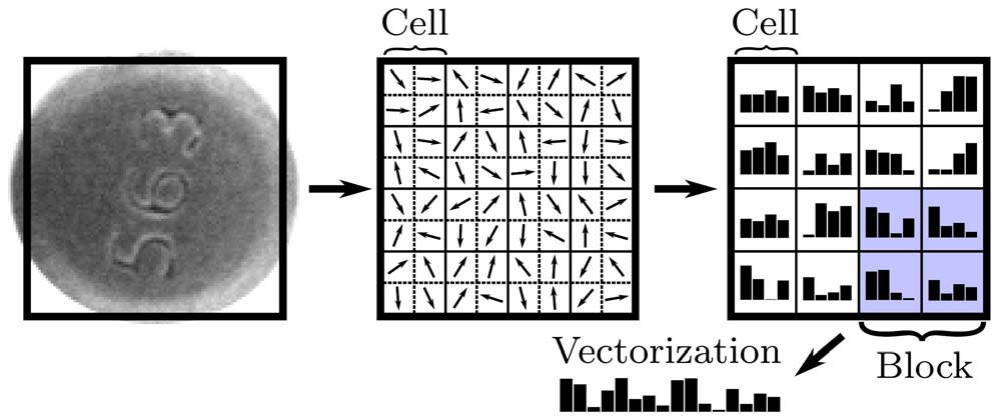
Histograms of Oriented Gradients. The detection window is subdivided into spatial cells, which are then concatenated into larger blocks.

**Figure 4:**
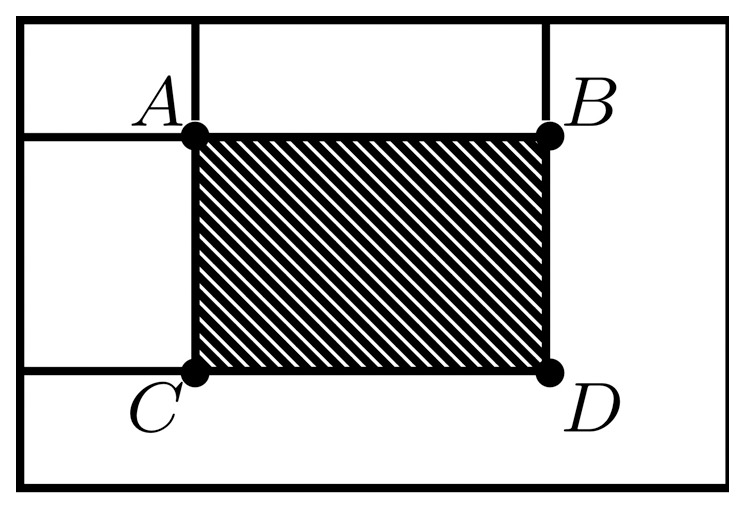
Integral image. The value 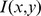 at any point 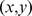 is the sum of all the pixels above and to the left of 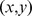 inclusive, 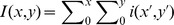 — integral 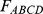 of pixel values within arbitrary rectangle 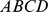 is obtained by 
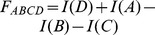
.

Dalal and Triggs [10] proposed Histograms of Oriented Gradients as a feature set for robust human detection and localization. A feature set describes local appearance and shape by distributions of gradient orientations. The basic implementation defines an object boundary (detection window), sub-divided into smaller spatial regions (cells) (Figure 3). Each cell contains a distribution of gradient orientations. Illumination invariance is achieved by normalizing the cell values over larger blocks, which are formed by grouping cells in a sliding fashion. Zhu et al. [11] extended the original work to non-uniformly tiled cells with varying cell sizes and block layouts. Extracting features from overlapping cells by a straightforward implementation is time consuming because we redundantly evaluate the same areas multiple times. To avoid this, Zhu et al. [11] proposed an integral image approach to HOG feature extraction. Integral images (Figure 4) enable a constant time HOG calculation over an arbitrary rectangular region.

### Reference set

**Figure 5:**
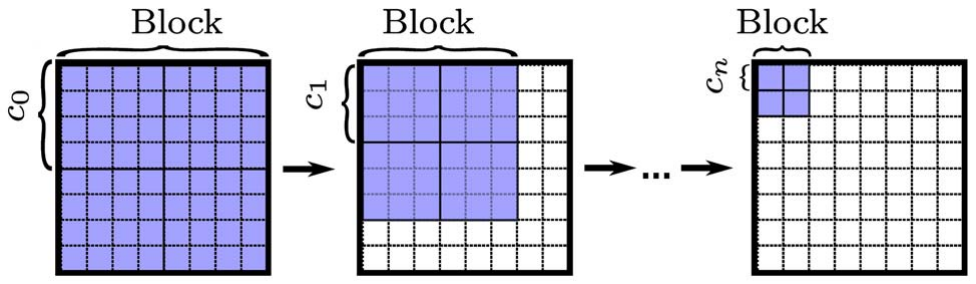
Initial set of cells. Cells are constructed by uniformly tiling the detection window with cells of decreasing size. Starting with whole detection window 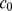, we iteratively decrease the size by a constant factor 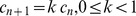.

We begin with the construction of a set of blocks 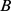 over which we calculate HOG features. Each block consists of 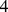 cells in a 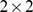 layout. To minimize user input, the blocks in a set 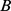 span over multiple scales. On each scale, the blocks uniformly tile the detection window. We start at the largest scale – the whole detection window – and then iteratively reduce the size by a constant factor until we reach the smallest scale (Figure 5).

With a set of blocks 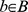 and a reference image 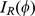, a reference set 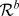 is constructed for each block 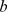.

Reference set 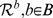, where 
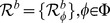
 is a set containing the features extracted from a block 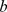 over all valid angles 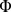. A set of valid angles 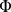 is obtained with discretization of the valid rotation interval. The dimensionality of a feature vector 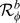 corresponds to the number of cells in each block and the number of histogram bins in each cell.

For all the angles 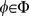, we rotate the reference image by 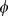 then calculate the HOG features over all blocks in 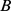. The resulting features are stored in corresponding reference sets 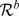.

#### Reference set adjustment

Initial reference sets, containing feature vectors describing an object appearance at all angles 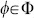, are constructed from a single image. This representation is biased, because it is constructed from a single reference image 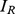 assuming a rotationally invariant appearance. Bias is evident in a case of a corrupted reference image or in a case of rotationally non-symmetric illumination. The assumption of a constant surface appearance is relaxed by a semi-supervised [12] self-training approach. This enables the accommodation of an angle-dependent surface appearance, assuming only a piecewise constant surface appearance. By that we mean that on an arbitrarily small interval 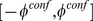 the surface appearance remains constant.

The semi-supervised self-training approach is performed in an iterative fashion. Reference sets 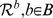 are iteratively adjusted, with a set of 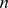 unlabeled training images 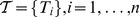. Starting with the initial reference sets, we estimate angles 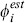 for all training images 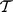. For each training image 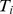, we construct another set in HOG space 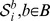, similar to the initial reference set 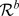, except that here each set describes the appearance only on the local interval 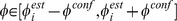.

**Figure 6:**
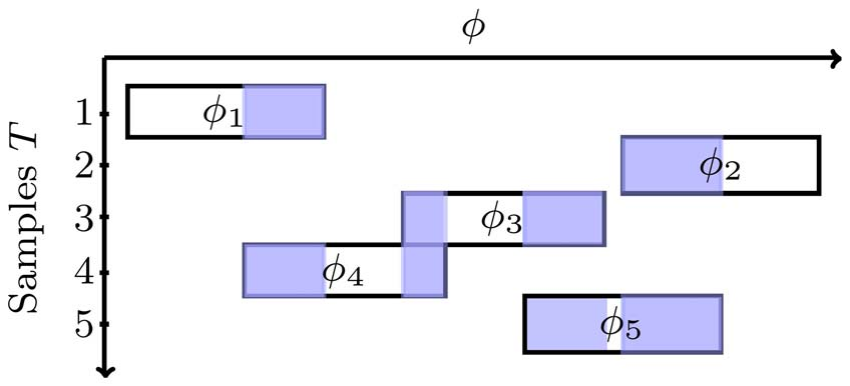
Reference set adjustment. Each of the training samples contributes to the reference set on local interval 
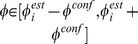
. For illustration purposes, angles with multiple representations are colored. Acquisition of unlabeled training images is usually trivial, thus an arbitrary number of feature representations can be obtained at every angle.

This way we obtain multiple sets 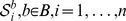, each containing a feature representation 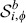 of an object on a local interval (Figure 6).

Note that each element of a reference set 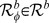 contains a feature representation of the corresponding block 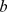 at the specific angle 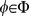. Each element 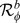 is replaced with the median value of all object representations 
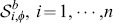
 at the specific angle 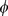. The whole adjustment procedure is iteratively repeated until the angle estimations of the training images remain stationary or the maximum number of iterations is reached.

### Weight estimation

**Figure 7:**
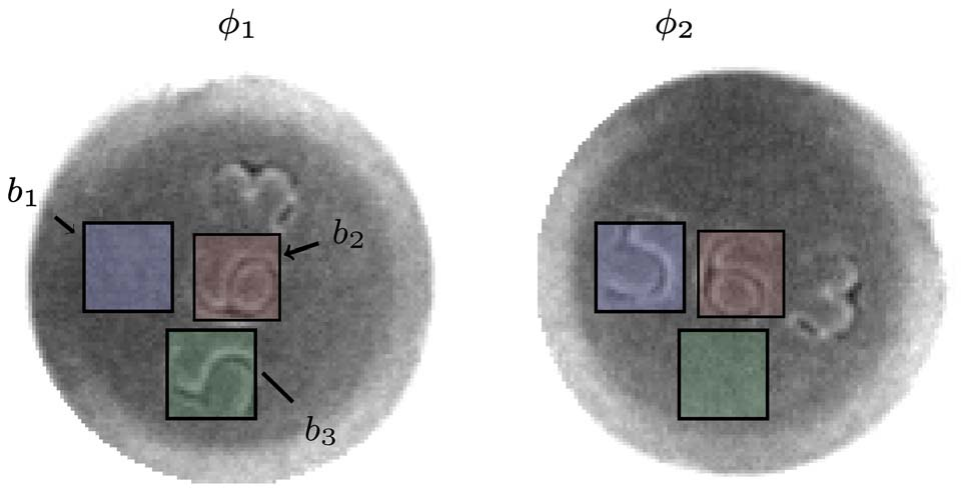
Set of blocks is equal for all rotation angles 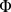. Each block contains relevant information, i.e., imprint, only at certain angles.

In addition to the iterative reference set adjustment, we introduce an angle-dependent block-weighting scheme due to an empirical observation that not every block is equally relevant at all rotation angles (Figure 7). Weights quantify the importance of the local region 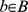 when voting for the angle 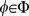.

Let 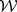 be a set containing weights 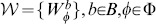 for all the blocks B at all valid angles 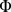. The weight 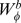 of block 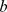 and angle 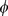 is estimated with an evaluation set 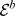, which is constructed by combining sets 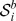 described in the previous section.

Each element in a set 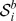 has a known angle 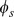 on interval 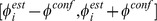 and represents an object appearance in the HOG feature space. The evaluation feature set for each block 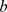 is a combination of all local reference sets: 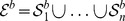.

For each element in a set 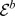 with a known angle 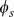, k nearest neighbors are located in the reference set 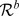. Each nearest neighbor in the reference set has a corresponding angle 
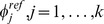
. The probability 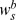 that an element 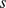 in 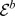 contributes to the true angle 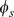 is a percentage of nearest neighbors in the 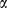 radius:
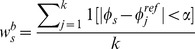
(2)


The radius 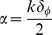 is calculated from the sampling resolution 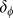 of the reference set and a selected number of nearest neighbors 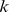. Essentially, 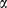 is chosen such that at most 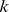 neighbors fit in the 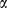 radius.

A weight 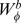 is obtained by collecting and averaging evaluation results 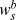 for all the elements in 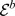 with the angle 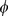:
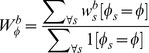
(3)


**Figure 8:**
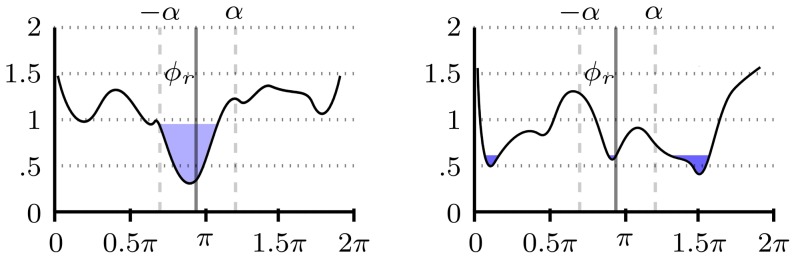
Nearest neighbors in an 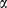 radius. Left, all 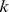 nearest neighbors are in an 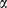 radius, by contrast most of the 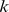 nearest neighbors on the right figure are spread over the interval 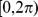.

This criterion should give higher weights to blocks that have a distance function with a well-defined global minimum. Figure 8, shows two distance functions. If the distance function 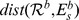 — which is a Euclidean distance — has a well-defined global minimum (Figure 8a), most of the 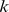-nearest neighbors will be in the 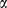 radius, resulting in high 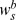. On the other hand, if a distance function does not have a well-defined global minimum (Figure 8b), 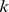 nearest neighbors will be spread over all the angles 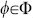, resulting in low 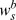.

### Rotation estimation

The rotation angle of the sample image 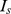 is estimated by approximate nearest-neighbor searches over the constructed reference sets 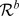. First, feature vectors over all the blocks 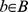 are extracted from the sample image 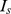. Then, for each feature vector corresponding to the block 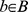, k nearest neighbors 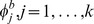 are located in the reference set 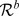. Each nearest neighbor contributes a single weighted-vote for its estimated angle.

All the votes are accumulated in an accumulator 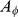 and weighted according to the weights in 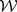:
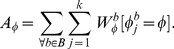
(4)


The estimated sample's angle 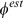 is then the angle with most accumulated votes:
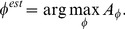
(5)


## Experiments and Results

### Datasets

**Figure 9:**
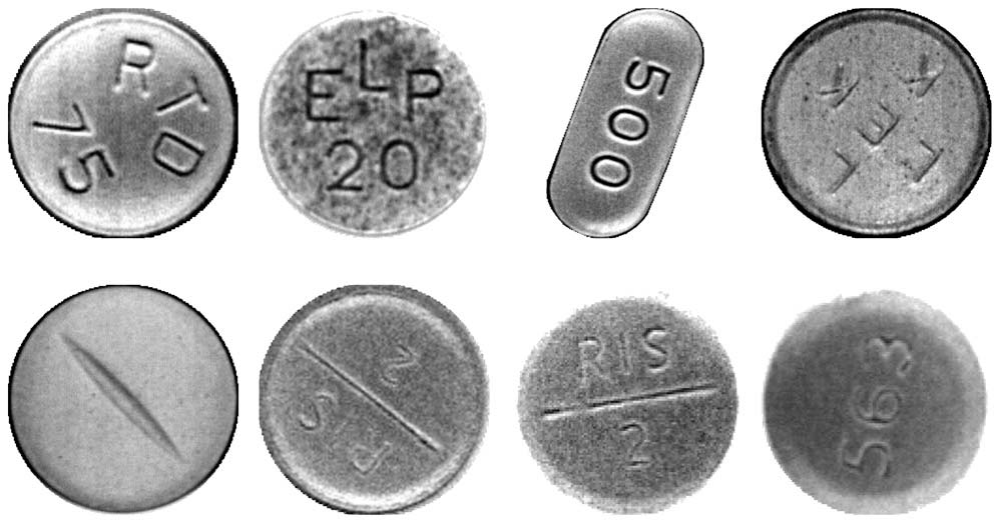
Evaluation datasets 1–8.

**Figure 10:**
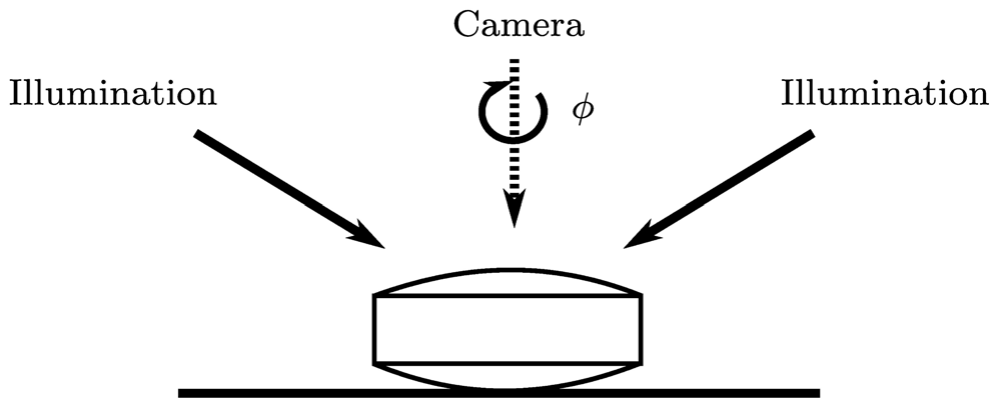
Illumination and camera view geometry. Camera view is parallel to the rotation axis.

Eight datasets of various image quality and imprint visibility were obtained and captured for evaluation (Figure 9). Datasets were captured with a SPINE machine vision system (Figure 1), using a trilinear line-scan camera and fixed white LED illumination. The setup is shown in Figure 10. The eight datasets contain 514, 419, 272, 383, 447, 407, 329, and 329 images, respectively. Tablets differ in size, color, shape, and imprint.

Datasets 7 and 8 contain the same tablet type but were captured under a different illumination angle. Datasets 1–5 contain tablets with good imprint visibility; by contrast, datasets 6–8 contain images with poor imprint visibility, i.e., the angle is often hard to determine even upon close examination.

All datasets with supporting information are freely available upon request.

### Implementation details

The proposed method along with the CPM method was implemented in C++. The HOG features were extracted using an integral image approach, due to blocks spanning over multiple scales of the detection window. An angle resolution 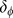 of the reference set was set to 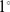. The same resolution was used for the similarity measure evaluation of the CPM. This effectively put the upper bound to estimation accuracy of both methods to 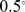. A nearest-neighbor search in the reference set was performed using the FLANN [13] library and its kd-tree [14] approximate nearest-neighbor implementation. A separate kd-tree was constructed for each block, effectively speeding up the nearest-neighbor search by an order of magnitude, compared to an exhaustive search. For each block, 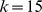 nearest neighbors were located, weighted, and stored in the vote accumulator 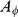. The accumulator was smoothed with a 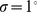 Gaussian kernel. The rotation angle was estimated with an exhaustive search over the vote accumulator. In the training phase, 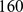 unlabeled training images were used with a confidence interval 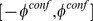, 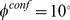.

### Metrics

The registration robustness and accuracy were evaluated by the criteria proposed by Špiclin et al. [6]. The registration robustness was determined by the percentage of successful registrations, i.e., registrations with an absolute error 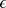 below 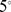. Estimation accuracy is defined as the mean absolute angular error of successful registrations. Using this definition, inaccuracy has the upper bound at 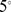. We found such restriction necessary to reduce the effect of outliers (results with large error) on the resulting accuracy. Otherwise, a single sample could have a disproportionally large effect on the resulting accuracy. Nevertheless, we quantified samples with large errors (samples with error 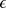 above 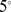) with hit percentage. A 5 degree upper bound was chosen to correspond with the upper bound proposed by Špiclin et al. [6].

An absolute error 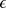 is a distance between the estimated angle 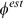 and the gold standard angle 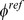:
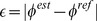
(6)


The reference angle 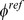 is estimated from 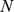 manually positioned corresponding points on the reference 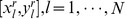 and sample 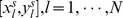 image. 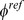 is estimated by minimizing the mean squared distance, between the corresponding points as a function of rotation:
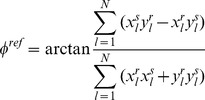
(7)


### Evaluation

The evaluation was performed with a one-round cross-validation. From each dataset, 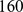 unlabeled images were used for the training, and the rest of the images were used for the evaluation. First, we evaluated robustness, accuracy, and speed on eight captured datasets. Then, we evaluated the registration robustness of the proposed method on synthetically degraded samples. Synthetic degradation allowed for evaluation at a predefined degree of surface degradation. Finally, we evaluated the effect of the reference set adjustment and weighting of the nearest neighbor estimators (nn-estimators) on accuracy and hit percentage.

#### Evaluation on real datasets

Table 1 shows the registration accuracy and the hit percentage for the eight captured datasets. Average execution times for each method are shown in Table 2. Both the proposed method and the CPM method successfully estimated the angles, that is with error 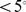, for all the samples in sets 3 and 5. For sets 1, 2, and 7, the CPM method failed on 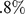, 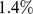, and 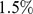 of samples, respectively. The proposed method, on the other hand, successfully estimated the angles for all the samples. Sets 4, 6, and 8 were more difficult datasets due to poor imprint visibility and 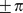 ambiguity. For set 4, the CPM method failed in 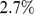 of cases and for set 8, in 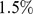 of cases. The proposed method successfully estimated the angles for 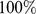 of the samples. For set 6, a success rate of the CPM is 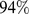. The samples in this set had a poor imprint visibility (Figure 9) and a near symmetric imprint shape. For some samples, the rotation angle was hard to determine even upon close examination. Irrespective of this, the proposed method exhibited a near 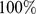 success rate.

**Table 1:** Registration results for the proposed method and two reference registration methods.

	Set 1	Set 2	Set 3	Set 4	Set 5	Set 6	Set 7	Set 8
**Error** (  )								
CPM	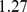	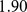	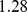	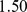	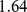	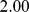	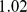	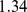
Proposed method	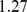	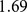	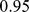	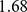	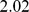	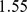	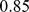	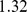
**Hits (** 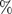 **)**								
CPM	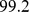	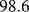	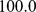	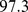	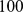	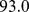	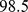	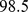
Proposed method	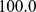	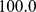	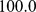	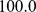	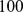	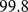	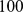	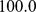

**Table 2:** Average process time for compared methods.

	Process time [ms]
CPM	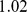
Proposed method	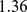

**Figure 11:**
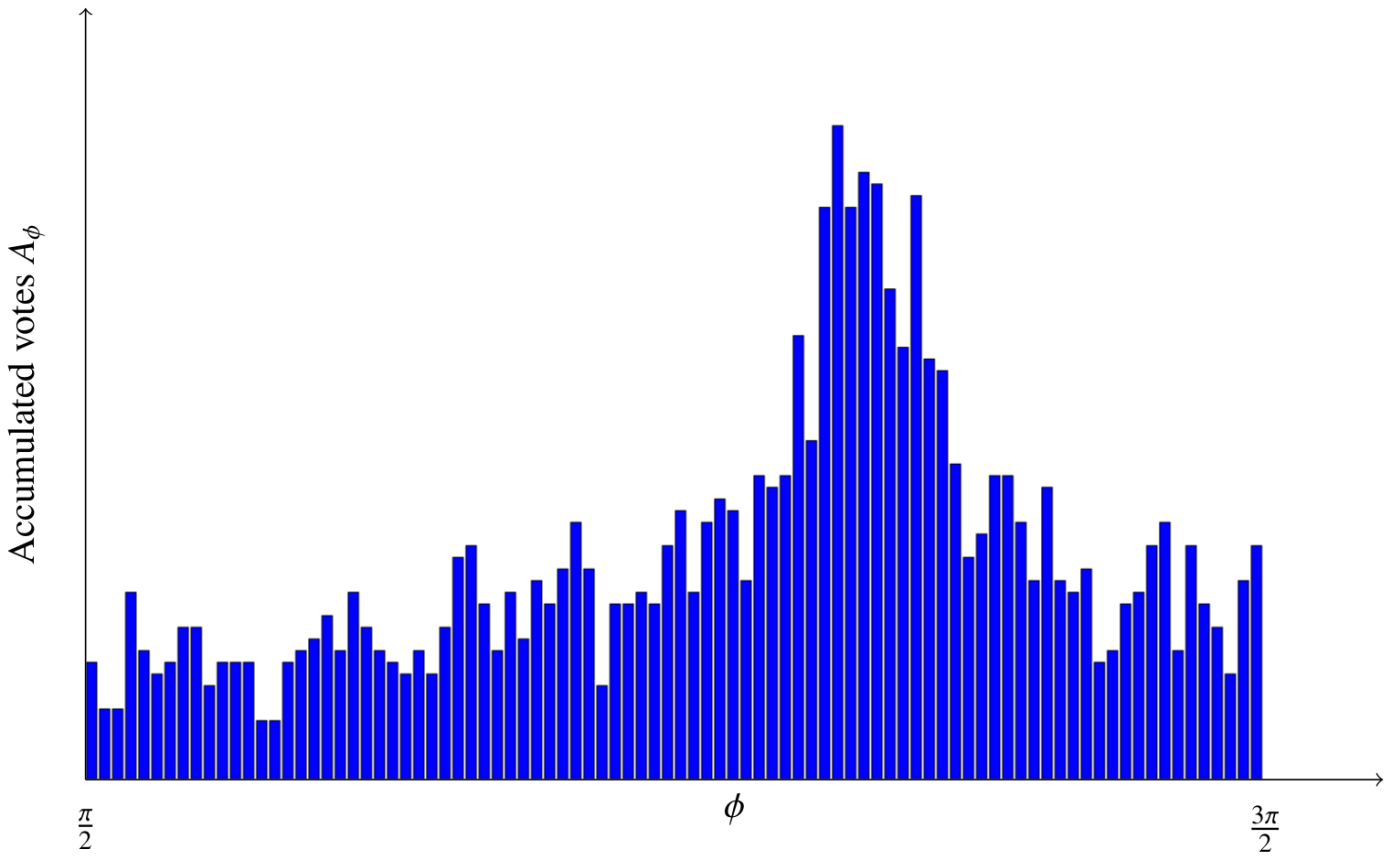
Distribution of accumulated votes is skewed. For illustration purposes only an interval 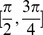 is shown.

The accuracy of the proposed method was comparable on all evaluation datasets (Table 1). For sets 1, 2, 3, 6, 7 and 8, the proposed method achieved the highest accuracy; while for sets 4 and 5 the difference in accuracy was negligible (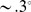). The accuracy of the proposed method was affected by accumulator smoothing. When smoothing the accumulator with the Gaussian kernel, we assumed that the votes were normally distributed with the mean at the true rotation angle. As shown in Figure 11, the vote distribution was skewed, thus smoothing introduced an estimation bias. Smoothing was, nevertheless, essential because of the limited number of votes.

#### Robustness evaluation

Two synthetically degraded datasets simulating defective surfaces were created by occluding a certain percentage of the tablet area. Various degrees of occlusion simulate various degrees of imprint defects. Datasets created in this way enable registration robustness and accuracy evaluation at a predefined degree of appearance changes.

**Figure 12:**
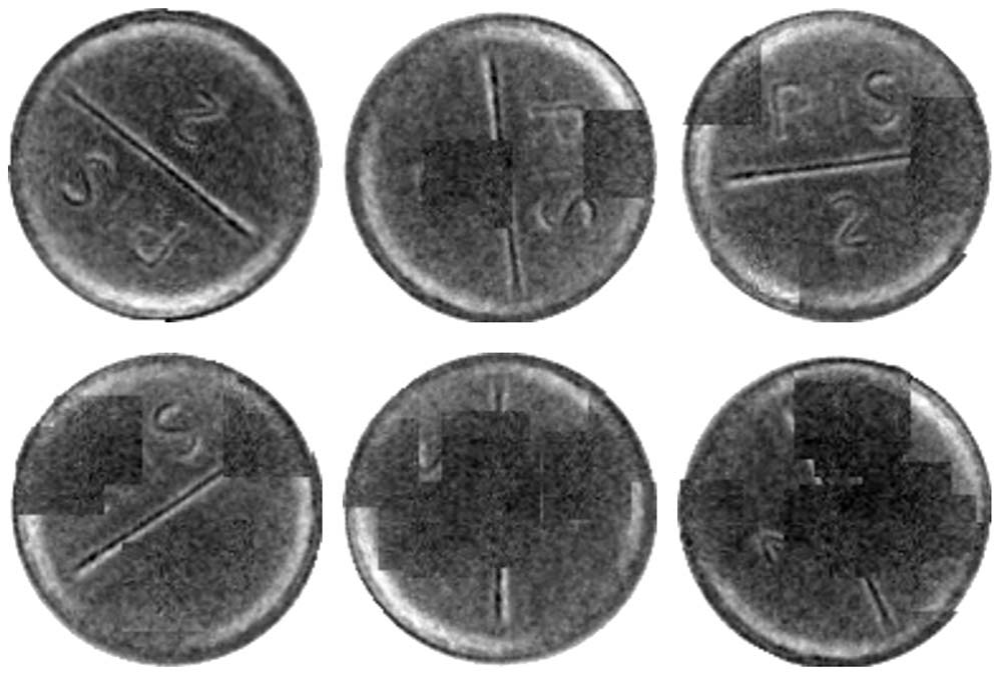
Synthetically degraded dataset. Occlusion patches are randomly spread over the tablet surface. Figures show samples with 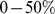 of the area occluded.

The first set was generated by degrading samples in dataset 1 and the second set, by degrading samples in dataset 6. The tablet area on each sample was occluded at random positions with patches taken from the tablet area without imprint (Figure 12).

**Figure 13:**
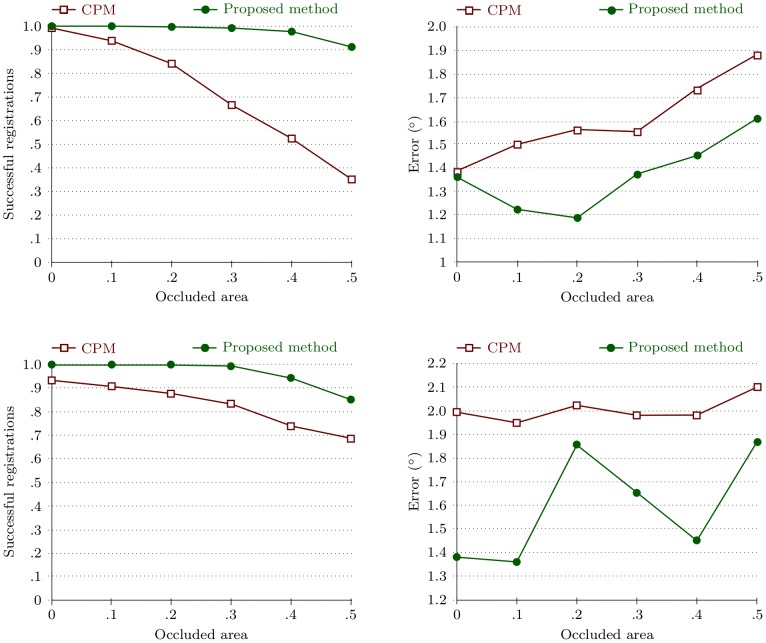
Robustness (left column) and accuracy (right column) evaluation, performed on synthetically degraded images. Results for first and second synthetically degraded datasets are presented in the first and second row respectively.

The evaluation of the first synthetically degraded dataset (Figure 13) showed that the success rate of the CPM declined to 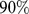 at 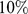 of the sample area occluded. The proposed method achieved a 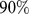 success rate even with 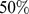 of the tablet area occluded. The evaluation of the second degraded dataset demonstrated similar results. Registration robustness is of utter importance when performing a model-based visual quality inspection, because a failed registration will result in wrong classification.

#### Effect of reference set adjustment and weighting of nn-estimators

The effect of the reference set adjustment and weighting of nn-estimators was evaluated on dataset 6; this set was used because it contained samples with high appearance variability and poor imprint contrast. Results are shown in Table 3. The results show that reference set adjustment is essential to increase registration robustness. The registration success rate without weighting and reference set adjustment was 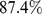. By introducing weights, the success rate increased to 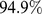; by adjusting the reference set, it increased to 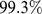. Combining the adjustment of the reference set and the weighting of the spatial regions, we obtained a 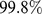 success rate. On this dataset, the CPM method achieved a 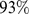 success rate.

**Table 3:** Effect of block weighting and reference set adjustment on registration accuracy.

	Uniform weights	Non-uniform weights
**Error** (  )		
Initial	1.52	1.51
Adjusted	1.59	1.55
**Hits (** 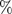 **)**		
Initial	87.4	94.9
Adjusted	99.3	99.8

## Discussion

The main difficulty in the registration of pharmaceutical tablets arises from intra- and inter-tablet variability. Furthermore, a registration method should be general enough to work for a variety of shapes, imprints, and surface appearances, with minimal human intervention, while retaining sufficient accuracy, robustness, and performance for real-time inspection (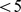 ms). A method based on the extraction of circular profiles [6] had been proposed previously. Its main advantages were simplicity and speed, given the assumption of rotationally symmetric illumination and rotationally invariant object appearance. It required a parameter fine-tuning for each tablet type individually, an accurate segmentation, and an accurate center estimation (estimation error below 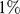 of an object size).

In practice, perfect diffuse illumination is undesirable for the inspection of surface structures, because shapes and surface variations are difficult to recognize. Under directed illumination, on the other hand, surface appearance varies with the angle of rotation.

In the proposed method – without reference set adjustment – a single reference image is used to build a reference set containing an object representation for all valid rotation angles. However, under directed illumination, a single reference image is not a good description of the surface appearance at all rotation angles, because appearance varies with the angle of rotation. The proposed method automatically adjusts a representative model of the object, avoiding the rotationally invariant appearance requirement. This is done by a semi-supervised self-training approach, iteratively improving the object representation at various angles. Due to a non-parametric approach to angle estimation this is easily incorporated into the estimation. The method builds the object representation assuming a locally constant appearance on an arbitrarily small interval 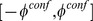. As already noted in [6], in-plane rotations of round tablets are random and uniformly distributed on 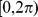 interval. Given the uniform distribution, we obtain 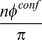 different feature representations at each angle. Here 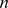 is the number of training samples. Clearly, an arbitrary number of feature representations can be obtained for an arbitrarily small 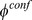 by increasing the number of training samples. This is a feasible approach, because the acquisition of a large set of unlabeled samples is usually simple to do and economical. An improved representation of an object is the main reason for the improvement of registration robustness.

Registration error increases slightly with reference set adjustment. This is because we use a finite number of unlabeled training samples for the reference set adjustment. To incorporate information of each unlabeled sample, a rotation angle for each sample must first be estimated using the unadjusted reference set. Consequently, we are adjusting the reference set with training samples with the estimated rotation angles, where some estimations can be erroneous. Erroneous estimations affect the adjustment process and subsequently introduce error into the estimation framework.

**Figure 14:**
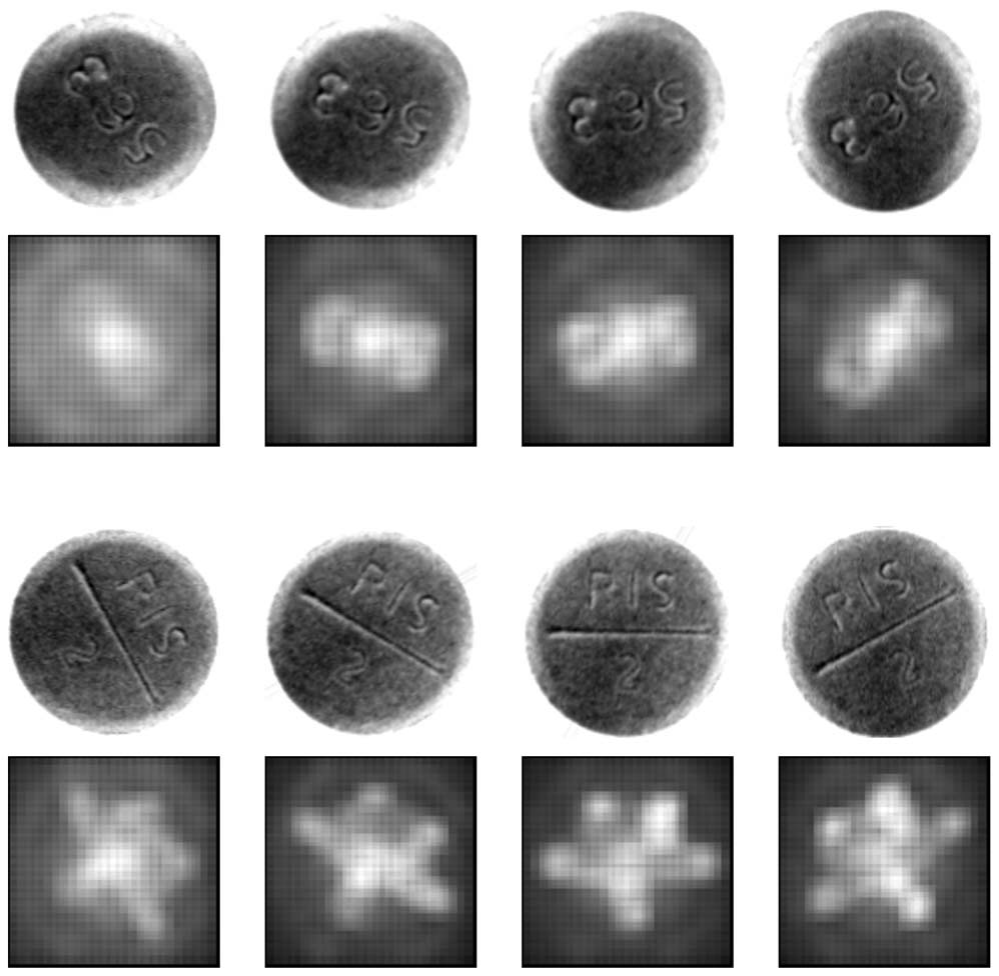
Cell weight visualization for different rotation angles. Higher intensity values represent higher weights.

By decoupling spatial regions and by treating each as an individual model, the dimensionality of each model is reduced, consequently enabling the use of spatial structures for a fast nearest-neighbor search. Decoupling also increases estimation robustness by eliminating the effects of outliers but reduces the discriminant power of each model. This approach closely resembles the description of the ensemble methods given in [15]. The advantage of the ensemble approach is shown by increased robustness and accuracy [16]. In the proposed method, each model determines an angle solely from its local neighborhood. This increases the robustness but reduces the discriminative power of each model. This reduction is alleviated by adjusting the reference set to represent better an object at all rotation angles, and by weighting each spatial region according to the angle for which it votes for. Figure 14 illustrates the weight of each spatial region at various angles for two different tablet types. Essentially, both weighting and reference set adjustment achieve a similar goal by different means. Both increase the robustness of the method by reducing the power of irrelevant spatial regions. To highlight the advantage of the proposed method further, note that all the results are obtained with the same set of parameters, whereas the CPM method requires parameter fine-tuning for each dataset individually.

In the proposed method, we used a uniform grid partitioning to calculate the HOG feature descriptors. Nonetheless, other partitioning strategies are also possible. For natural images, Zhou et al. [17] partitioned an image into several rectangular horizontal and vertical partitions. On natural images, many objects remain in vertically and horizontally consistent locations relative to other objects; note that this does not necessarily hold true for other types of images. In [11], Zhu et al. used the Adaboost algorithm to select an optimal set of blocks for human detection. In our work, we did not consider this approach, because boosting algorithms [18], while common for feature selection, usually require extensive training times and large training sets.

In the context of the current state-of-the-art methods for rigid-body registration, the proposed method uses a dense grid for feature calculation, with a finite set of possible transformations. This approach imposes an upper bound to registration accuracy. By contrast, most state-of-the-art methods start by detecting local regions invariant under affinity, and then calculate features over those regions. Several region detectors were proposed for this task [19], mostly focusing on region detection on planar structures. A descriptor [20] is associated with each region, which is used to establish correspondences between the regions and their descriptors on multiple images. Region detection and correspondence search are difficult in the presence of repetitive or undescriptive structures. With a dense approach, both region detection and correspondence search are avoided, thus eliminating two possible points of failure. This is possible because we are concerned only with a single degree of freedom – not the whole affine transformation. Note, that in medical imaging numerous robust methods [21] have been proposed for rigid [22] [23] and non-rigid [24] registration. However, the application of those methods in industrial setting is usually impractical due to high computational complexity.

## Conclusion

In this paper, we proposed a new rotation estimation method and demonstrated its performance and efficiency relative to another method for rotation estimation of pharmaceutical tablets. The proposed method requires minimal human intervention; all evaluation results were obtained with the same set of parameters. In addition, it does not require segmentation, because the voting scheme reduces the voting power of outliers, e.g., wrongly segmented or defective regions. Using a real dataset, we show that introducing unlabeled training images increases the robustness by relaxing the constant appearance assumption. Despite the fact that the evaluation focused on pharmaceutical tablets, we consider the method useful for any application that requires robust real-time rotation estimation.
